# Carbonization of a stable β-sheet-rich silk protein into a pseudographitic pyroprotein

**DOI:** 10.1038/ncomms8145

**Published:** 2015-05-20

**Authors:** Se Youn Cho, Young Soo Yun, Sungho Lee, Dawon Jang, Kyu-Young Park, Jae Kyung Kim, Byung Hoon Kim, Kisuk Kang, David L. Kaplan, Hyoung-Joon Jin

**Affiliations:** 1 Department of Polymer Science and Engineering, Inha University, Incheon 402-751, Korea; 2 Carbon Convergence Materials Research Center, Korea Institute of Science and Technology, San 101 Enha-ri, Bongdong-eup, Wanju-gun 565-905, Korea; 3 Department of Nano Material Engineering, Korea University of Science and Technology, 217 Gajeong-ro, Yusung-gu, Daejeon 305-350, Korea; 4 Department of Materials Science and Engineering, Seoul National University, Seoul 151-742, Korea; 5 Department of Physics, Incheon National University, Incheon 406-772, Korea; 6 Department of Biomedical Engineering, Tufts University, Medford, Massachusetts 02155, US

## Abstract

Silk proteins are of great interest to the scientific community owing to their unique mechanical properties and interesting biological functionality. In addition, the silk proteins are not burned out following heating, rather they are transformed into a carbonaceous solid, pyroprotein; several studies have identified potential carbon precursors for state-of-the-art technologies. However, no mechanism for the carbonization of proteins has yet been reported. Here we examine the structural and chemical changes of silk proteins systematically at temperatures above the onset of thermal degradation. We find that the β-sheet structure is transformed into an *sp*^2^-hybridized carbon hexagonal structure by simple heating to 350 °C. The pseudographitic crystalline layers grew to form highly ordered graphitic structures following further heating to 2,800 °C. Our results provide a mechanism for the thermal transition of the protein and demonstrate a potential strategy for designing pyroproteins using a clean system with a catalyst-free aqueous wet process for *in vivo* applications.

## Abstract (web summary)

The strength and stability of silk proteins is thought to be related to the high content of β-sheets within their structures. Here, the authors show that when heated at high temperature, and above that of thermal degradation, these β-sheets are transformed into an ordered hexagonal graphitic structure.

## Graphical abstract

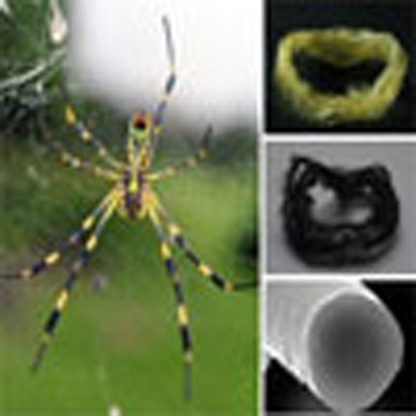

Proteins are biological macromolecules linked by peptide bonds of various amino acids with their own characteristic composition, which results in the arrangement of an amino sequence and the formation of the distinctive secondary structures, such as α-helices, β-sheets and turns (or loops). Among them, β-sheet conformation is the most stable secondary structure organized by numerous assemblies of interchain hydrogen bonding between the adjacent peptide blocks, and known to be associated with interesting functions in protein materials, for example, unique mechanical properties of natural protein fibres, such as spider silk and worm silk^1^,^2^,^3^ and irreversible protein misfolding/aggregation causing devastating human diseases, such as Alzheimer's and Parkinson's diseases^4^,^5^. Over the past decades, considerable effort has been directed towards understanding the β-sheet system and adapting various applications, such as the synthesis of high performance fibres using the robustness^6^, a resist for electron-beam lithography^7^ and a substrate for bioelectronics^8^ using the controlled water stability according to the β-sheets crystal contents. As another interesting feature, some β-sheet-rich proteins such as *Nephila clavata* spider dragline silk (spider silk) and *Bombyx mori* silk (worm silk) are not fully decomposed, and remain as carbonaceous forms^9^,^10^,^11^. When sufficient heat is applied in an inert atmosphere, pyrolysis of organic polymers begins by breaking down the C–C bonds of the polymer backbone. The pyrolysis process for a polymer chain may proceed in three ways^12^,^13^. First, the chain may degrade into small fragments and vaporize as H_2_, CO and CO_2_ gases, together with hydrocarbons such as CH_4_, C_2_H_4_ and C_2_H_6_. Second, the chain may collapse to form aromatic molecules, which may stack to form a lamellar plastic phase. Third, the chain may transform into a conjugated carbon structure without forming a plastic phase, creating a carbonaceous solid termed ‘char'. Which of these processes occur depends on the heat treatment temperature (HTT). Thus, the pyrolytic behaviour of silk proteins without aromatic backbones or crosslinked structures suggests that the protein molecules were restructured to form unsaturated or aromatic structures during pyrolysis.

Here we examine changes in the structural and chemical configurations of silk proteins systematically at temperatures above the onset of thermal degradation (that is, *T*>250 °C). We find that β-strands are transformed into an *sp*^2^-hybridized carbon hexagonal structure with pseudographitic crystalline layers from the β-sheet structure following heating to 350 °C, and the carbon basic structural units (BSUs) from β-sheet structure grew to form highly ordered graphitic structures following further heating to 2,800 °C. We provide a mechanism for the thermal transition of the protein and the graphitization behaviour. Moreover, our results demonstrate the potential for designing pyroproteins using a clean system with catalyst-free and aqueous-based wet process.

## Results

### Pyrolysis behaviour of silk proteins

**Figure 1:**
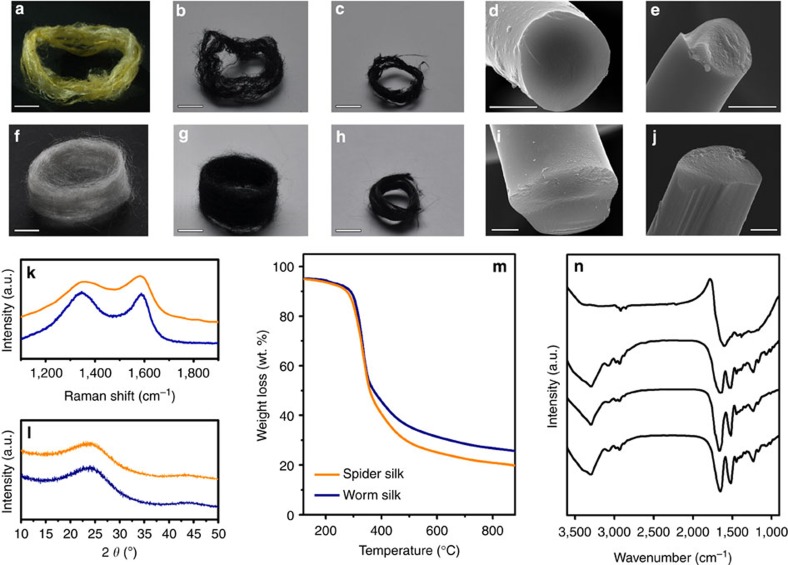
Pyrolysis behaviour of silk proteins. Photographs of (**a**) dragline silk fibres obtained from *N. clavata* spider webs and after pyrolysis at (**b**) 800 °C and (**c**) 2,800 °C. Scanning electron microscopy (SEM) images of dragline silk fibres after pyrolysis at (**d**) 800 °C and (**e**) 2,800 °C. Scale bars, 5 mm for **a**–**c** and 2 μm for **d**,**e**. Optical images of (**f**) silk fibroin fibres produced by *Bombyx mori* silkworm and after pyrolysis at (**g**) 800 °C and (**h**) 2,800 °C. SEM images of silkworm silk fibres after pyrolysis at (**i**) 800 °C and (**j**) 2,800 °C. Scale bars, 5 mm for **f**–**h** and 2 μm for **i**,**j**. (**k**) Raman spectra and (**l**) XRD patterns of the pyrolysed spider silk (orange) and silkworm silk (navy blue). (**m**) Thermogravimetric analysis curves of spider silk (orange) and silkworm silk (navy blue). An inert nitrogen gas atmosphere was used, and the scan rate was 10 °C min^−1^. (**n**) FT-IR spectra of an untreated silk protein and of silk protein samples with HTTs in the range 250–350 °C.

As shown in Fig. 1a–j, silk protein fibres (see Supplementary Fig. 1) may be transformed into a carbonaceous material by heating at 2,800 °C under an inert atmosphere. The G and D bands of the Raman spectra, which occur at ∼1,350 and ∼1,580 cm^−1^, respectively, are indicative of such a transition, as well as the broad graphitic (002) peak around 2*θ*=24° in X-ray diffraction (XRD) patterns, which originates from the conjugated carbon structure (see Fig. 1k,l). In spite of a reduction in the length and diameter of the fibres (see Fig. 1a–j and Supplementary Fig. 2), both spider silk and worm silk maintained their fibrous morphologies following pyrolysis at 800 °C, with quite high residual masses of ∼23.4 and 33.2 wt%, respectively, as shown in Fig. 1m. These results suggest that the silk protein is affiliated with a char-type polymeric precursor without melting. This pyrolysis behaviour differs from other types of natural protein-rich materials or amide-linked polymers, such as soybean, egg-white, sheep wool or nylon 6; however, chicken breast, which is a β-sheet-rich protein, exhibited similar behaviour, with a carbon residue percentage of ∼22.1 wt% following pyrolysis at 800 °C (see Supplementary Fig. 3). The pyrolysis behaviour of β-sheet-rich proteins with no aromatic backbone or crosslinked structures indicates that the protein molecules are restructured to form a conjugated system following pyrolysis.

The thermal degradation of silk proteins initiates at temperatures around 250 °C, as shown by the thermogravimetric analysis results (Fig. 1m). The critical chemical changes in silk proteins take place at temperatures in the range 300–350 °C, whereby the inherent characteristic peaks of polypeptides around 1,620, 1,520, 1,230 and 3,040 cm^−1^ vanish (these features are attributed to amide I (that is, C=O stretching vibrations), amide II (C–N stretching vibrations), amide III (N–H deformation vibrations) and N–H stretching vibrations^14^, respectively), as well as an absorption peak at ∼1,590 cm^−1^, which is attributed to C=C or C=N skeletal vibrations in aromatic compounds^15^, and can be seen in the Fourier transform infrared spectroscopy (FT-IR) data shown in Fig. 1n.

### Structural and chemical transitions of silk proteins

**Figure 2:**
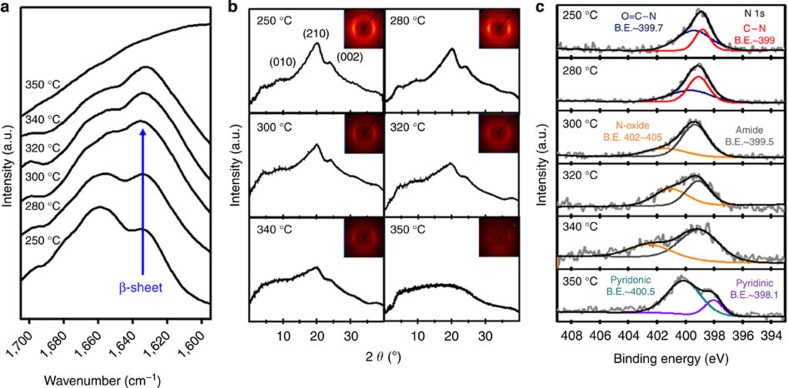
Structural and chemical transitions of proteins rich in β-sheets during pyrolysis. (**a**) Amide I band region of the FT-IR spectra for silk proteins heated to temperatures in the range 250–350 °C for 60 min. Each spectrum was acquired in absorbance mode by accumulating 32 scans. (**b**) Wide-angle XRD patterns of silk proteins heated to various temperatures. Characteristic peaks of the β-sheet crystals were evident at 9.34°, 20.80° and 25.78° corresponding to (010), (210) and (002) planes, respectively. (**c**) XPS high-resolution N 1s spectra for silk proteins heated to temperatures in the range 250–350 °C. The peak at 399.7 eV corresponds to O=C–N; the peak at 399 eV corresponds to C–N; the peak at 402–405 eV corresponds to N-oxide; the peak at 400.5 eV corresponds to pyridonic N; and the peak at 398.1 eV corresponds to pyridinic N.

More specific examinations were carried out using silk protein samples prepared by heat treatment at temperatures in the range 250–350 °C. The infrared spectral region 1,700–1,600 cm^−1^ was assigned to absorption due to the polypeptide amide I, which is commonly used to determine the secondary structures of silk proteins^16^,^17^. The absorption peak at 1,622–1,637 cm^−1^ is a characteristic of the β-sheet structure, whereas a random coil or α-helical structure results in a broad and indistinct peak in the amide I band region^14^. The characteristic peak intensity of the β-sheet conformation at ∼1,629 cm^−1^ grew as the HTT increased from 250 to 300 °C in the amide I band region (Fig. 2a). This behaviour is often observed with the conformational transition of silk proteins from unstable random coil structures to a stable β-sheet crystal structure^17^,^18^,^19^,^20^. However, as shown in the thermogravimetric analysis curve in Fig. 1a,e, rapid mass loss of ∼27 wt% (∼43 wt% of the overall mass loss for worm silk) was observed within the temperature range 250–300 °C. For this reason, changes in the characteristic peaks for silk proteins may be caused by large decreases in the fraction of amorphous regions compared with β-sheet regions^20^, suggesting that the observed rapid mass loss occurred primarily due to thermal decomposition of the protein backbone in the amorphous region. Figure 2b shows XRD patterns that also support this analysis; the Bragg reflections at 9.6, 19.8 and 24.2°, which correspond to the (100), (210) and (002) planes of the protein β-sheet structure^21^, respectively, were clearly observed in silk protein samples heated to 250 and 280 °C. However, these Bragg reflections decreased markedly in the silk protein samples heated to 300 °C, which indicates that deformation of the β-sheet conformation is associated with the thermal degradation of the silk protein at temperatures in excess of 280 °C. The FT-IR spectra of the silk protein samples heated to 300, 320 and 340 °C exhibit clear characteristic peaks of the β-sheet conformation, despite the significant mass loss; however, this characteristic peak vanished when the HTT reached 350 °C. In addition, the XRD patterns reveal that the characteristic peaks for β-sheet crystal structure gradually weakened and broadened as the HTT increased from 300 to 340 °C, and disappeared following heating to 350 °C, indicating that the β-sheet crystals progressively decomposed at temperatures in the range 300–340 °C, and were destroyed at 350 °C.

Figure 2c shows X-ray photoelectron spectroscopy (XPS) data, which reveal a chemical transition between 250 and 350 °C, as well as the formation of new aromatic compounds at 350 °C. We find two distinct peaks in the N 1s spectra of samples heated to 250 °C: one centred at 399.7 eV and one at 399.0 eV. These correspond to O=C–N and C–N bonds, respectively, and are similar to the spectra of raw silk proteins^22^, indicating that there was no particular transition of the chemical structure below 250 °C. Similar analysis of the silk protein sample heated to 280 °C also reveals two distinct peaks that correspond to amide bonds, whereas the relative intensity of the O=C–N peak was reduced, attributed to chain scission at the relatively weak C–N bond^23^. This finding indicates that no chemical species formed, even at temperatures that resulted in rapid degradation. In contrast, a peak corresponding to nitrogen-bonded N-oxide compounds appeared centred at 402–405 eV following thermal treatment at temperatures between 300 and 340 °C, as well as a peak corresponding to an amide bond centred at 399.5 eV; these features are indicative of the presence of peptide bonds and the formation of N-oxide compounds from the broken chains. When the HTT was 350 °C, the amide and N-oxide peaks of the N 1s spectra disappeared, and aromatic nitrogen species peaks centred at 398.1 and 400.5 eV appeared, which may be attributed to pyridinic and pyridonic nitrogen^24^. These FT-IR, XRD and XPS data suggest that the new chemical compounds are related to pyrolysis of the β-sheet-structured protein molecules, transforming the proteins into thermostable aromatic compounds. Indeed, silk protein treated at 350 °C for 3 h did not burn (see Supplementary Movie 1). The aromatization of the linear polymer backbone can be accomplished by an exothermic reaction such as cyclization or crosslinking; however, differential scanning calorimetry (DSC) curves of the silk proteins did not exhibit evidence of an exothermic reaction, rather an endothermic peak in the range 250–350 °C (see Supplementary Fig. 4). Considering the simultaneous reactions of degradation of polypeptide chains (an endothermic reaction) and the following transition into a ring structure of the resultant molecules (an exothermic reaction) during DSC, we may expect a very small net change in the overall energy. As a result, an exothermic reaction may be concealed in the DSC analysis by the presence of a larger endothermic reaction (see Supplementary Fig. 4 and Supplementary Note 1). A dramatic increase in the electrical conductivity between an HTT of 320 and 350 °C further supports the formation of a conjugated system (Supplementary Figs 5 and 6 and Supplementary Note 2).

### Development of carbon structure from silk proteins

**Figure 3:**
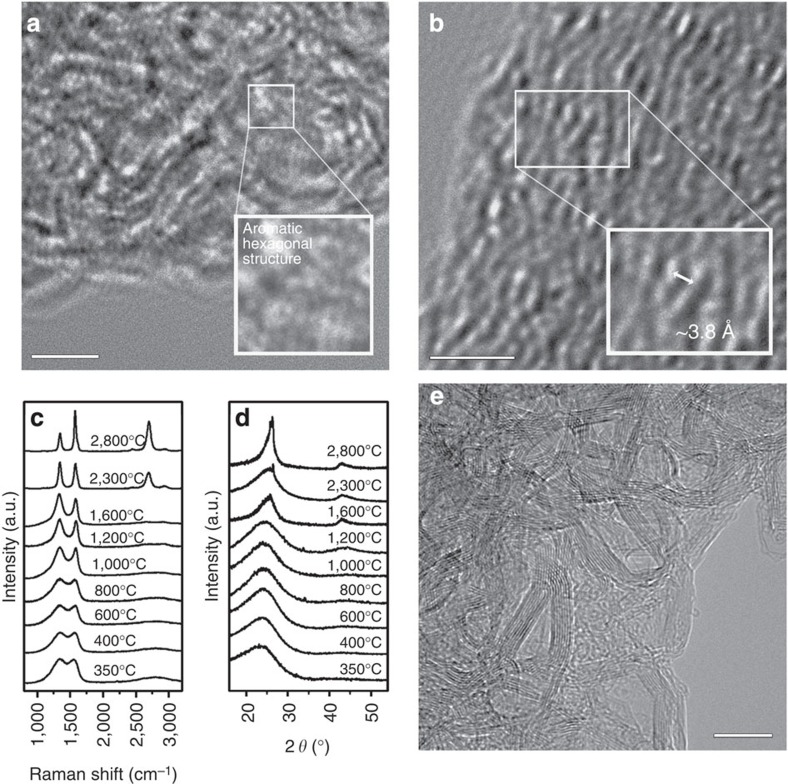
Formation and development of carbon microstructure from proteins. (**a**,**b**) Atomic-scale TEM images of the silk protein-derived carbon heated to 350 °C. Scale bars, 2 nm. (**c**) Raman spectrum and (**d**) XRD patterns of the silk protein-derived carbon as a function of the HTT. (**e**) FE-TEM image of a silk protein sample heated to 2,800 °C. Scale bar, 10 nm.

We performed microstructural analyses of the silk proteins that were heat treated at 350 °C. The amide bonded protein backbone disappeared, and a conjugated system was formed. Hexagonal aromatic rings with dimensions of ∼2.5 Å were observed using high-resolution transmission electron microscopy (Fig. 3a). In addition, an aligned polyhexagonal structure with nanometre dimensions (that is, BSUs^25^) along the fibre axis was observed (Fig. 3b, Supplementary Fig. 7 and Supplementary Note 3), which is similar to the β-sheet structure. This result strongly suggests that the aligned polyaromatic molecules, that is, BSUs, were formed from the β-sheet structure at a relatively low temperature of 350 °C. Raman spectra of the sample heated to 350 °C reveal a pseudographitic crystalline structure, with two distinctive carbon characteristic peaks centred at ∼1,350 (D band) and ∼1,580 (G band) cm^−1^, and XRD patterns show a band around 24.7°, which corresponds to the (002) plane of a graphitic structure^26^,^27^,^28^ (Fig. 3c,d). Using these Raman and XRD data, the lateral dimensions of the resulting BSU-carbon structure were found to be *L*_a_=1.6 nm, with an interlayer spacing of *d*_002_=0.38 nm. Further heat treatment of the pyroproteins resulted in a gradual change of the carbon microstructure (Fig. 3c,d, Supplementary Figs 8–10, Supplementary Note 4, and Supplementary Table 1). Hereafter, we denote the heat-treated pyroproteins as, for example, pyroprotein-400 having been heated to 400 °C. The integral intensity ratios of the D to G bands, that is, *I*_D_/*I*_G_, were ∼2.6 for pyroprotein-400, -600 and -800, with *L*_a_ ∼2 nm. For the pyroproteins heated to temperatures in the range 1,000–2,300 °C, the D and G bands become narrower, indicating the development of an *sp*^2^-hybridized carbon structures^29^,^30^. In particular, the Raman spectrum of pyroprotein-2,300 exhibited a two-dimensional peak centred at 2,691 cm^−1^, which corresponds to the formation of three-dimensional-ordered stacked graphitic layers^31^. As the HTT increased to 2,800 °C, a highly developed graphitic structure with an average interlayer distance of *d*_002_=0.338 nm and an average of *L*_*a*_=16 nm formed (Fig. 3c,d). These structures exhibited narrower two-dimensional bands compared with pyroprotein-2,300, with increased intensity and a shift toward 2,699 cm^−1^. This is indicative of a more developed graphitic stacked structure, and is consistent with the resulting large electrical conductivity of∼1,600 S cm^−1^ for pyroprotein-2,800 (Supplementary Fig. 11). The carbon microstructure can be controlled by tuning the β-sheet structure of the precursor materials (Supplementary Fig. 12). In addition, the carbon morphology and dimensions are controllable, and one-dimensional nanofibres, two-dimensional nanosheets and three-dimensional nanospheres can be formed (Supplementary Figs 13–15 and Supplementary Methods) from regenerated silk proteins using an aqueous solvent and a ductile heating system, without requiring the use of organic solvents or catalysts, which have issues with toxicity^32^. This suggests the potential for various applications, including bioelectronics and biomedical devices.

**Figure 4:**
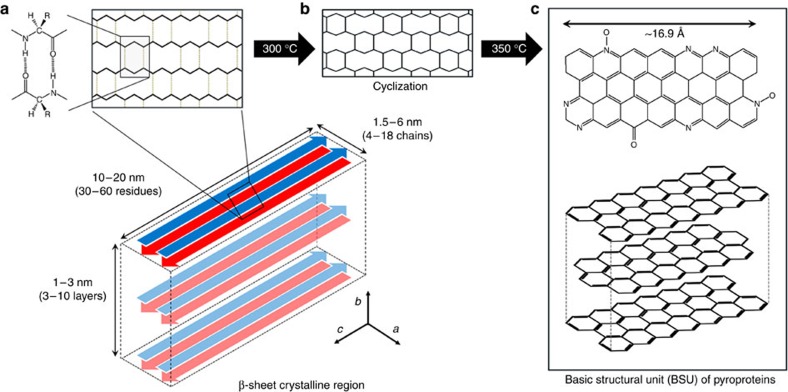
Schematic of β-sheet-derived carbon BSUs. (**a**) The structure of the hydrogen-bonded β-sheet. (**b**) At temperatures in excess of the onset of thermal degradation, the β-sheet structure is converted into a thermally stable aromatic structure via exothermic cyclization. (**c**) Following heating to 350 °C, the β-sheet structure is transformed into a stacked polyaromatic carbon structure.

Figure 4 shows schematic diagrams of models for the formation of the carbonaceous materials (that is, pyroprotein) following heat treatment of the β-sheet structure. A β-sheet structure is composed of two (or more) protein strands, and is stabilized by numerous hydrogen bonds between the amide proton and carbonyl oxygen of adjacent peptide chains (Fig. 4a). The aromatization of the linear polymer backbone may be accomplished via an exothermic reaction, such as cyclization or crosslinking. At temperatures in excess of 300 °C, cyclization or aromatization may result from intermolecular dehydration or condensation between the neighbouring molecular chains (Fig. 4b), leading to heteroaromatic BSUs (Fig. 4c). Furthermore, a β-sheet structure composed of peptides with small side chains, such as glycine or alanine, allows intersheet stacking to form a three-dimensional structure due to van der Waals interactions, resulting in the formation of stacked carbon clusters with a (002) plane.

## Discussion

In this work, we have investigated the pyrolysis of silk proteins from the onset of thermal degradation at 250 °C to general graphitization at 2,800 °C. The β-sheet structure of the polypeptides was gradually destroyed at temperatures between 300 and 340 °C and subsequently transformed to a conjugated *sp*^2^-hybridized hexagonal carbon structure following heating to 350 °C. The β-sheet structure aligned along the fibre axis resulted in a well-aligned formation of polyaromatic molecules, or BSUs. By further heating to 2,800 °C without any further chemical treatment and with no solvent or catalyst, the microstructure of the pyroproteins changed to a highly developed graphitic structure. In addition, control over β-sheet structure of the silk proteins resulted in different carbon microstructures, and nanocarbons with one-, two- and three-dimensional morphologies were fabricated from the regenerated silk proteins. This demonstrates that it is possible to fabricate carbon materials with controlled microstructure and morphology via a wet process.

## Methods

### Materials

Cocoons from *B. mori* silkworms were purchased from the Uljin Farm, South Korea, and boiled for 25 min in an aqueous solution containing 0.02-M Na_2_CO_3_ (99%; OCI Co.), and then rinsed thoroughly using deionized water to extract the glue-like sericins and other impurities. Following drying at room temperature for 72 h, the resulting fibrous material was used in further experiments. Spider dragline silk filaments produced by a *N. clavata* were collected directly from spider webs collected at Inha University campus (South Korea).

### Heat treatment

The silk protein samples with HTTs in the range 250–350 °C were prepared using a thermal analyser (NETZSCH STA 409 PC Luxx simultaneous thermal analyser, Germany). Approximately 20 mg of the silk protein sample was placed in an alumina crucible, which was 5 mm in diameter and 10 mm in height. The samples were heated to the desired temperature in a nitrogen atmosphere at a rate of 5 °C min^−1^. The silk protein samples with HTTs in the range 400–1,600 °C were carbonized in an alumina furnace. Following the removal of any water by heating to 150 °C for 2 h, the samples were heated at a rate of 5 °C min^−1^, followed by a 1-h isotherm at the desired temperature. A graphitization furnace (ThermVac, Korea) was used to achieve temperatures greater than 2,300 °C. After loading the samples into the furnace, the temperature was increased to 1,800 °C at 10 °C min^−1^, to 2,300 °C at 5 °C min^−1^ and to 2,800 °C at 3 °C min^−1^. An argon atmosphere was used for the samples heated to temperatures in excess of 400 °C (minimum purity, 99.9990%; gas flow, 100 cm^3^ min^−1^).

### Thermal transition analyses

FT-IR spectroscopy (VERTEX 80v, Bruker Optics, Germany) was carried out using the KBr disc method to obtain spectra in the range 500–4,000 cm^−1^ using 32 scans. The XRD patterns were recorded using a Rigaku DMAX 2500 using Cu-Kα radiation (wavelength *λ*=0.154 nm) with a step size of 0.02° and a step time of 1 s at 40 kV and 100 mA. To obtain meridional diffractograms, fibrous samples were pulled and fixed on a slide glass. XPS (PHI 5700 ESCA) was performed using monochromated Al Kα radiation (*hν*=1,486.6 eV).

### Carbon microstucture investigations

XRD patterns, Raman spectra and TEM were used to determine the microstructure of the silk protein-derived carbon structures as a function of the HTT. After fixing the sample on a slide glass using adhesive tape, Raman spectra were obtained over the range 800–3,500 cm^−1^ under ambient conditions using a laser excitation wavelength of 514.5 nm (giving a photon energy of 2.41 eV) with a power of 16 mW. The laser beam was focused using a × 100 objective lens, resulting in a spot size ∼1 μm in diameter. The acquisition time was 10 s, and the number of circulations was three. The morphology of the carbon materials derived from the silk protein fibres was observed using field emission scanning electron microscopy (S-4300, Hitachi, Japan) and transmission electron microscopy (FE-TEM) using a JEM2100F (JEOL, Japan) equipped with an energy-dispersive X-ray spectrometer. The samples were finely ground and dispersed in ethanol, and a drop of the resulting dispersion was deposited on a copper TEM grid.

## Additional information

**How to cite this article**: Cho, S.Y. *et al*. Carbonization of a stable β-sheet-rich silk protein into a pseudographitic pyroprotein. *Nat. Commun.* 6:7145 doi: 10.1038/ncomms8145 (2015).

## Supplementary Material

Supplementary Figures, Tables, Notes, Methods and References Supplementary Figures 1-15, Supplementary Table 1 Supplementary Notes 1-4, Supplementary Methods and Supplementary References

Supplementary Movie 1 Burning test of silk protein fibres heat treated at 350°C
