# Supplementary material for: Carbonization of a stable β-sheet-rich silk protein into a pseudographitic pyroprotein
Source: Nat Commun. 2015 May 20;6:7145. doi: 10.1038/ncomms8145 (PMC4455128; doi:10.1038/ncomms8145)
Supplement: Supplementary Figures, Tables, Notes, Methods and References — Supplementary Figures 1-15, Supplementary Table 1 Supplementary Notes 1-4, Supplementary Methods and Supplementary References [file ncomms8145-s1.pdf]

Supplementary Figures

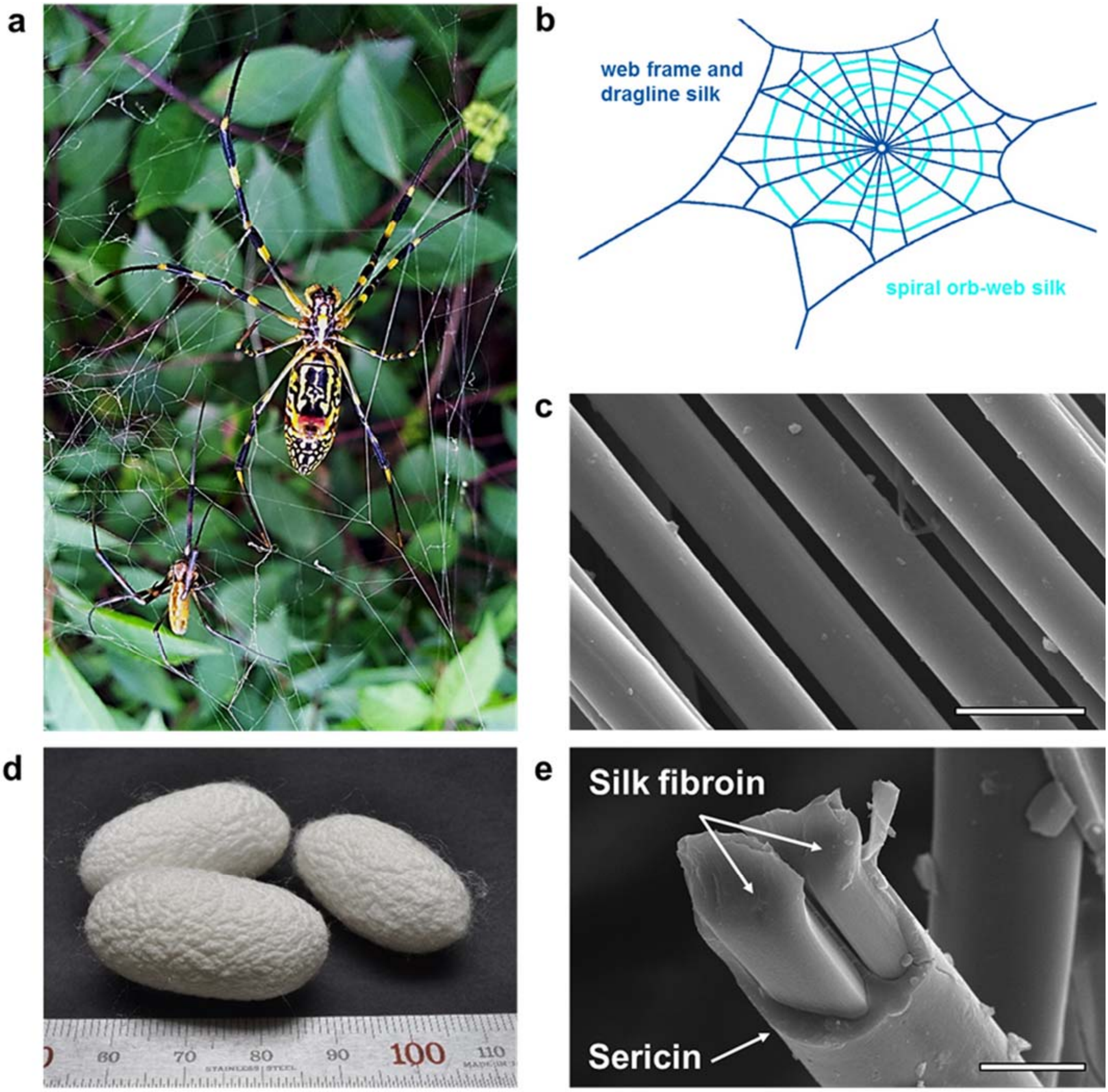

Supplementary Figure 1. Spider and worm silk fibres. (**a**) Optical photograph of *N. clavata* female (larger) and male (smaller) spiders on a web. (**b**) Schematic diagram of the spider web composed of a spiral orb-web supported by frame silk threads and draglines. (**c**) FESEM image of dragline silk fibres produced by the *N. clavata* spider, with an average diameter of 5.4 ± 0.4 μm. Scale bar, 10 μm. (**d**) Optical image of cocoons from the *B. mori* silkworm. (**e**) FESEM image of cocoon fibres that consist of the two fibrils of silk fibroin and a coating of sericin. Scale bar, 10 μm.

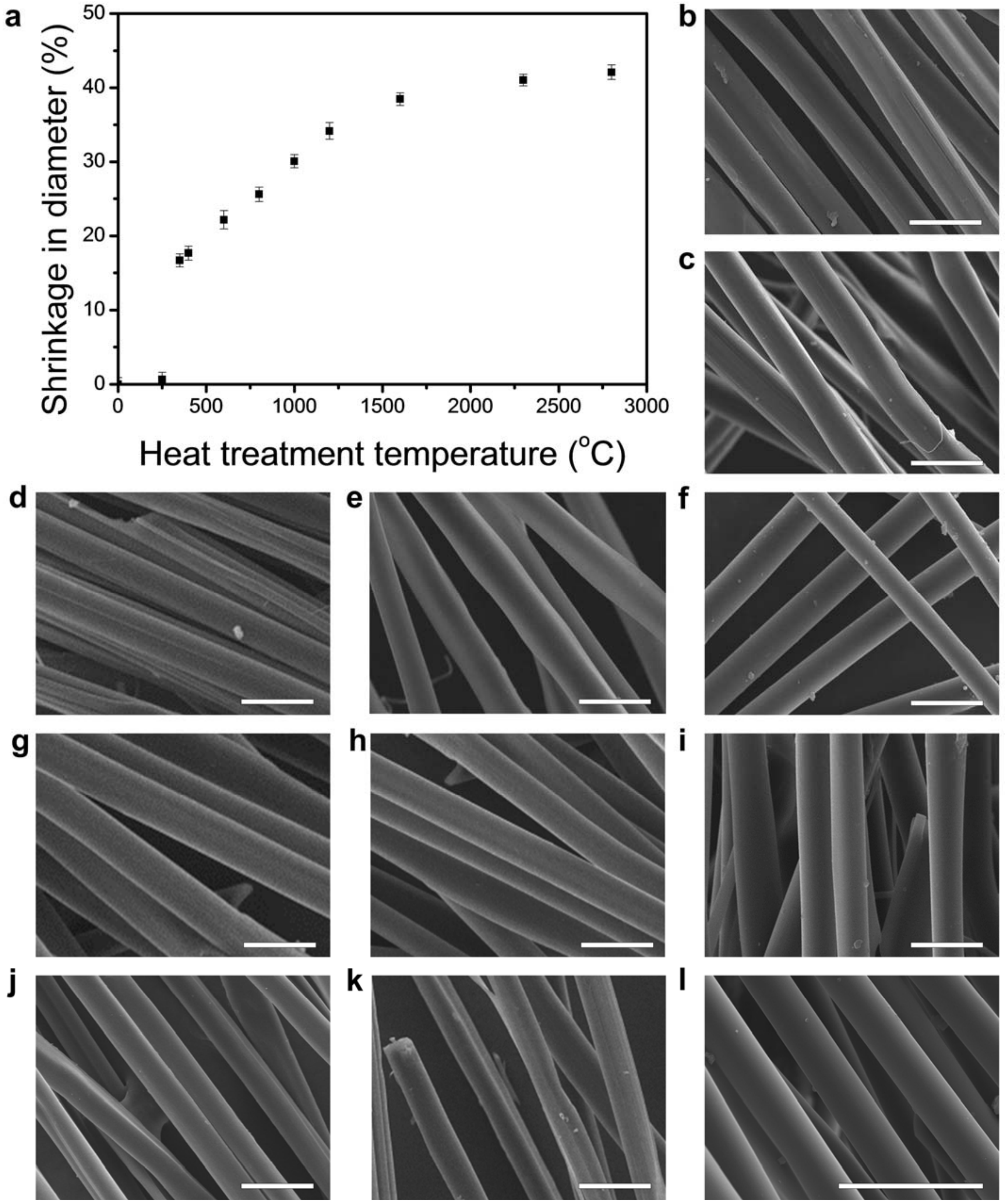

Supplementary Figure 2. (**a**) The change in diameter of the silk fibres as a function of temperature. FESEM images of (**b**) raw silk and silk fibres heated to (**c**) 350ºC, (**d**) 400ºC, (**e**) 600ºC, (**f**) 800ºC, (**g**) 1000ºC, (**h**) 1200ºC, (**i**) 1400ºC, (**j**) 1600ºC, (**k**) 2300ºC, and (**l**) 2800ºC. Scale bars, 20 μm.

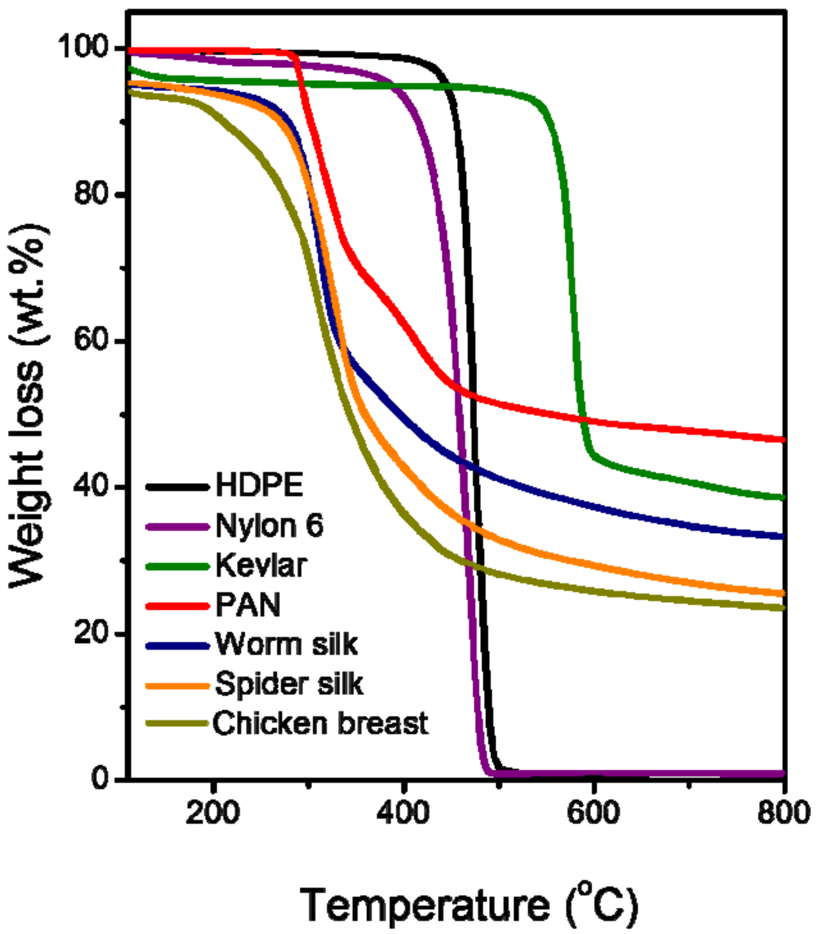

Supplementary Figure 3. TGA curves of diverse polymeric materials. A flowing nitrogen gas atmosphere was used, and the scanning speed was 10°C/min.

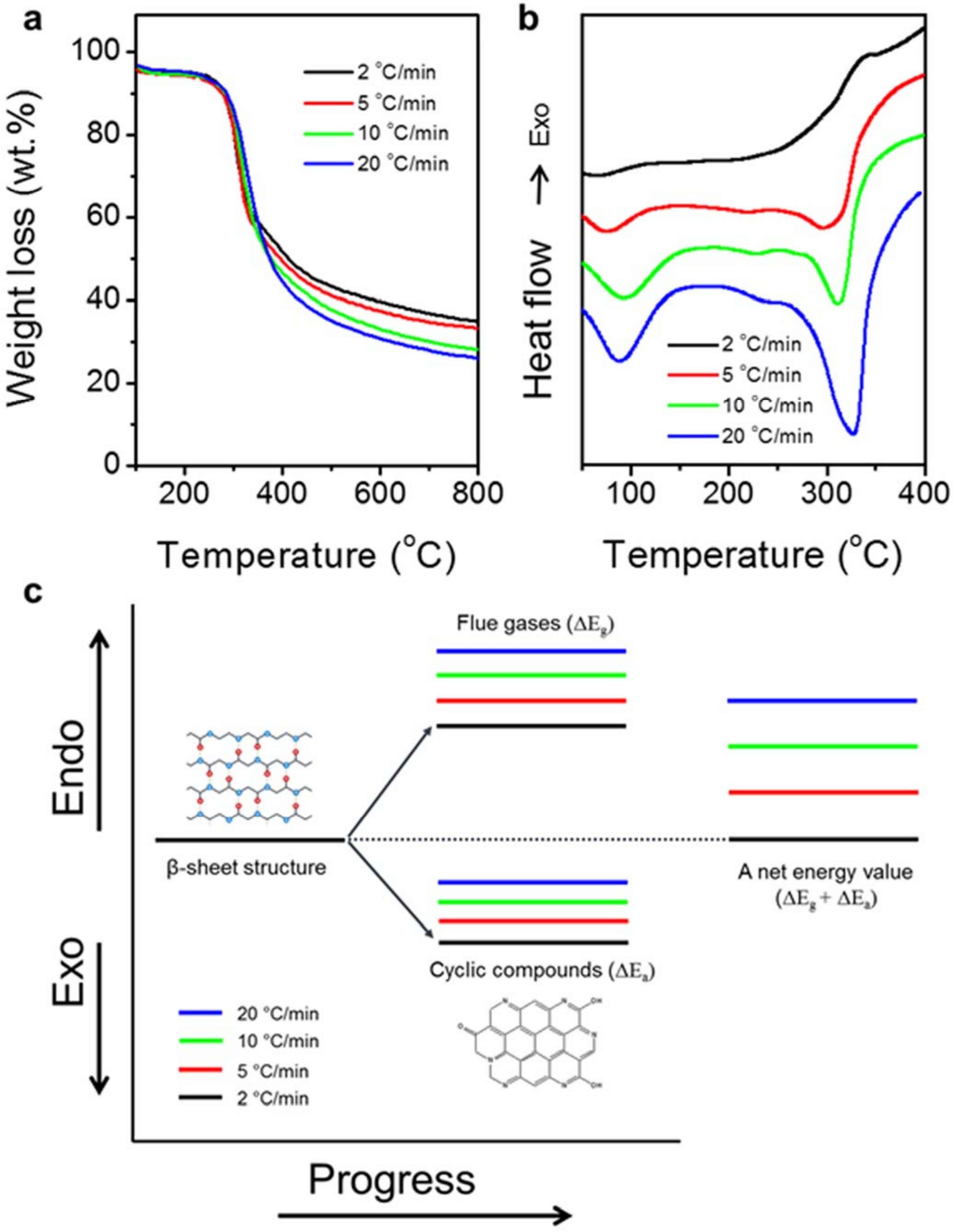

Supplementary Figure 4. (**a**,**b**) TGA and DSC analyses of silk protein with various heating rates. (**c**) An energy diagram showing the thermal degradation and transition of the protein backbone into a ring structure.

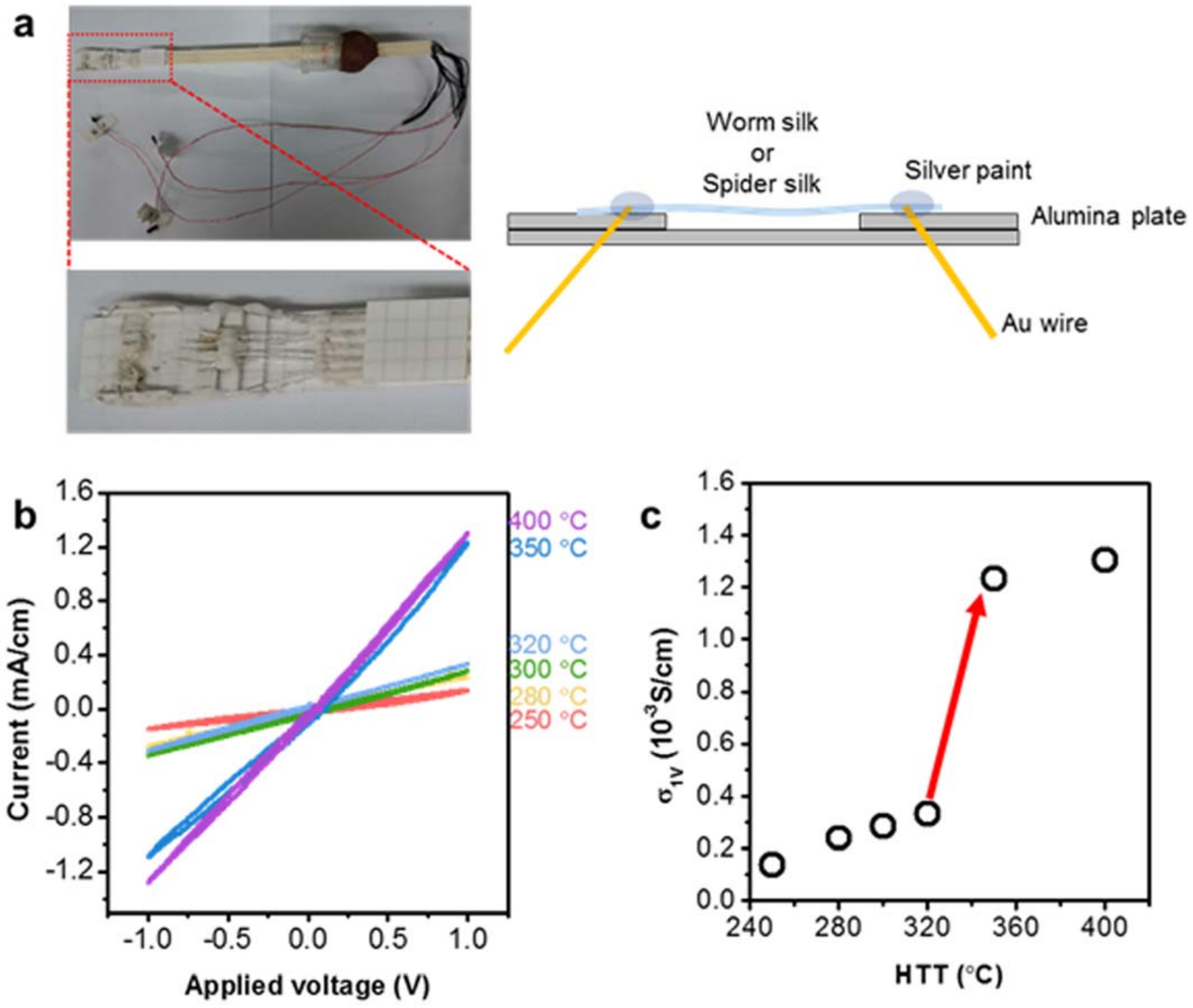

Supplementary Figure 5. Electrical properties of the silk fibres as a function of temperature. (**a**) The sample holder used for the measurements of electrical conductivity at high temperatures. (**b**) *I-V* curves of the worm silk fibres with various HTTs and (**c**) the conductivity obtained from the current at an applied voltage of 1.0 V as a function of the HTT.

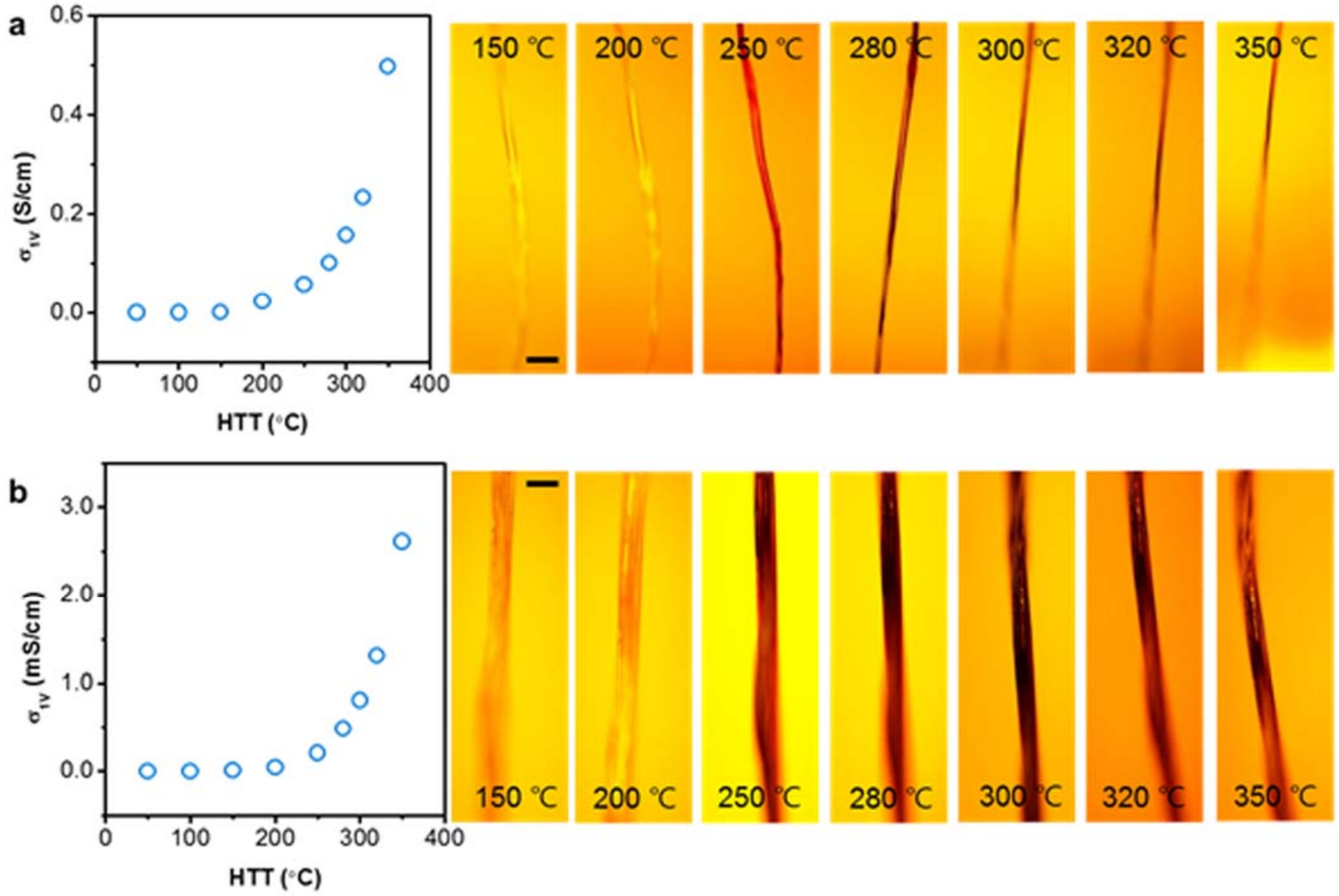

Supplementary Figure 6. **The conductivity σ_{1V} obtained from *in situ* measurements.** (**a**) Worm silk and (**b**) spider silk fibres. A change in colour was observed from 250°C in both optical images. The scale bar in the optical image is 100 μm.

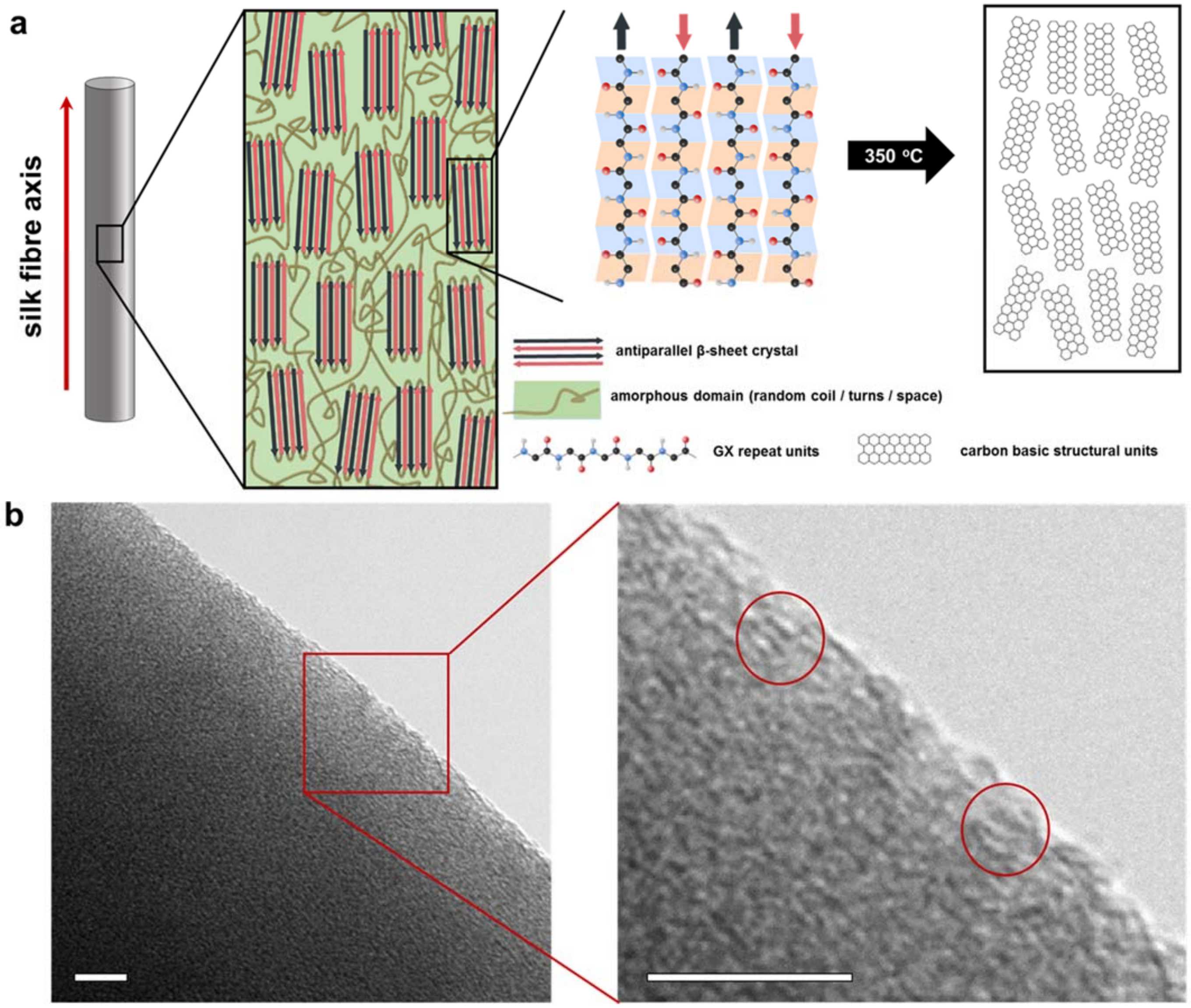

Supplementary Figure 7. The transition of a β-sheet structure into a carbon layer. (**a**) Schematic for the structural features of silk protein fibre consisting of amorphous domain and β-sheet crystals transformed into BSUs by thermal restructuring. (**b**) TEM images of silk protein fibre heat-treated at 350°C with different magnifications. Scale bars, 10 nm.

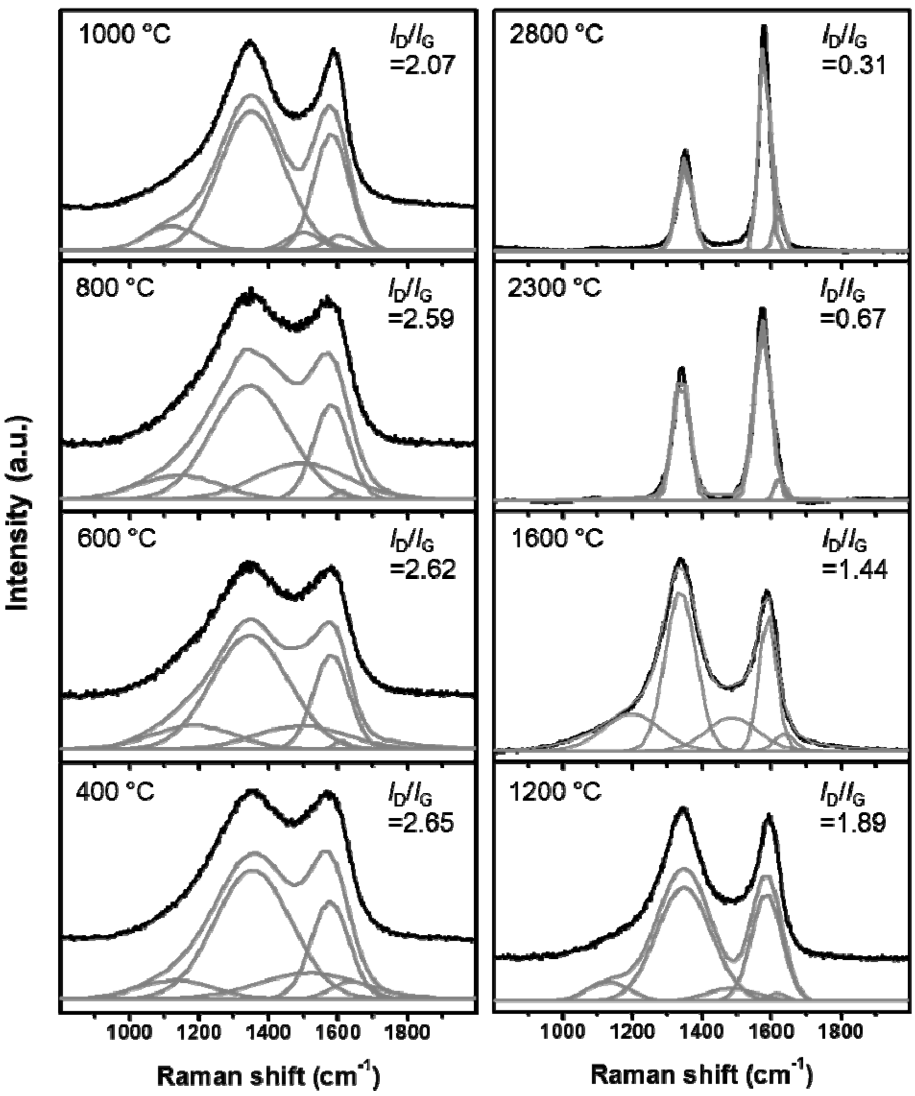

Supplementary Figure 8. Deconvolved Raman spectra of silk protein samples heat treated to temperatures ranging from 400 to 2800°C.

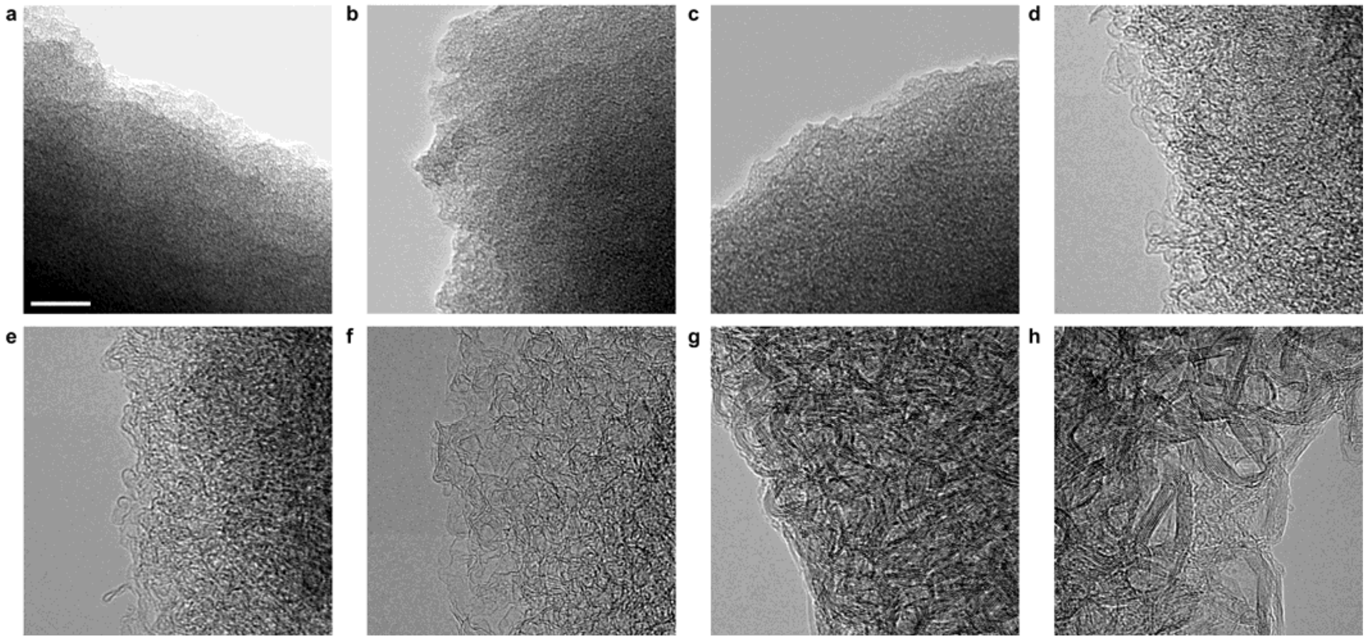

Supplementary Figure 9. FE-TEM images of the carbonised silk protein fibre at different HTTs. FE-TEM images of the silk-protein-derived carbon structures heated to (**a**) 400ºC, (**b**) 600ºC, (**c**) 800ºC, (**d**) 1000ºC, (**e**) 1400ºC, (**f**) 1600ºC, (**g**) 2300ºC, and (**h**) 2800ºC. The scale bar in **a** represents 10 nm and refers to **b-h**.

.

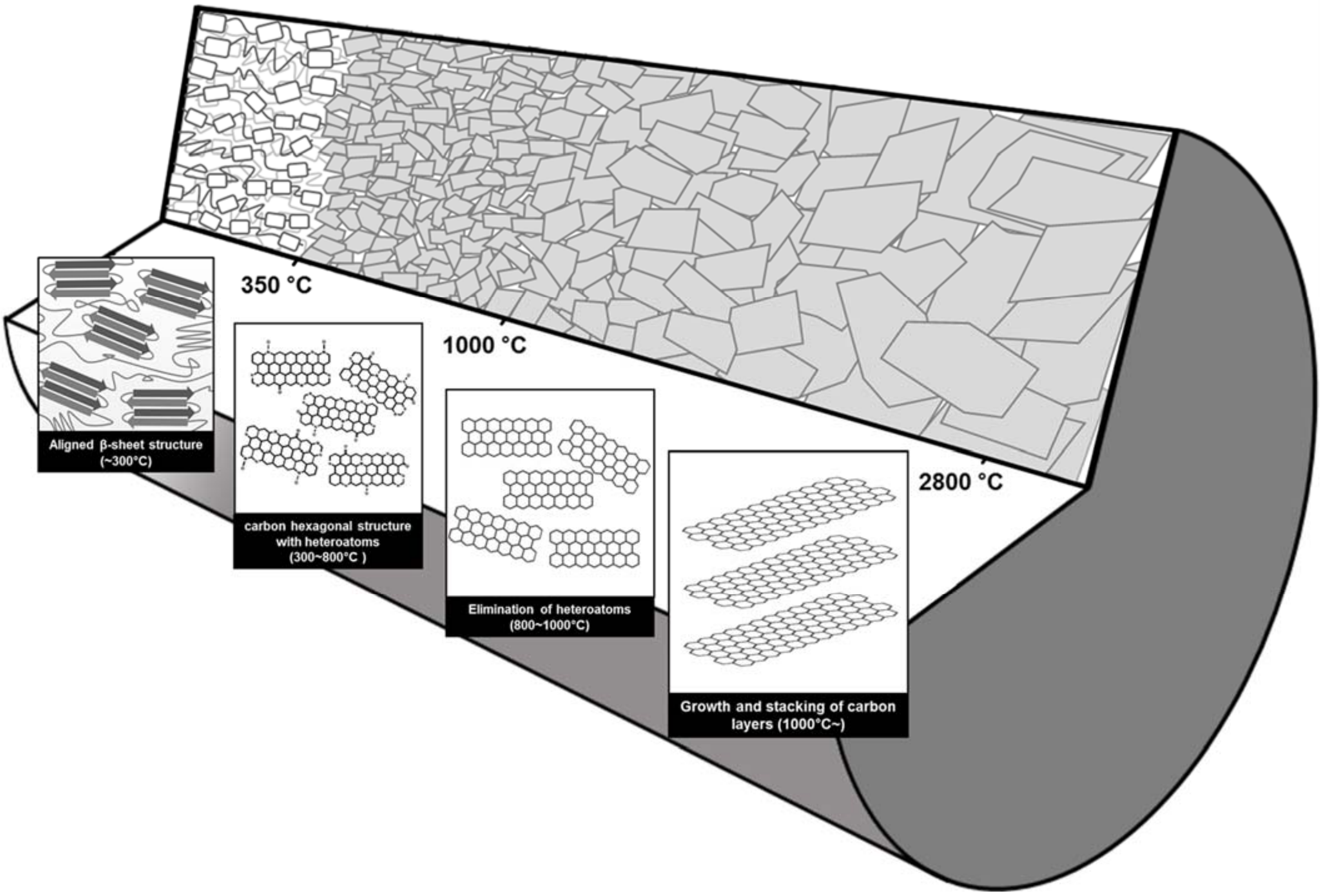

Supplementary Figure 10. Illustration for the development of carbon structure from silk protein with an increase of HTTs to 2800°C.

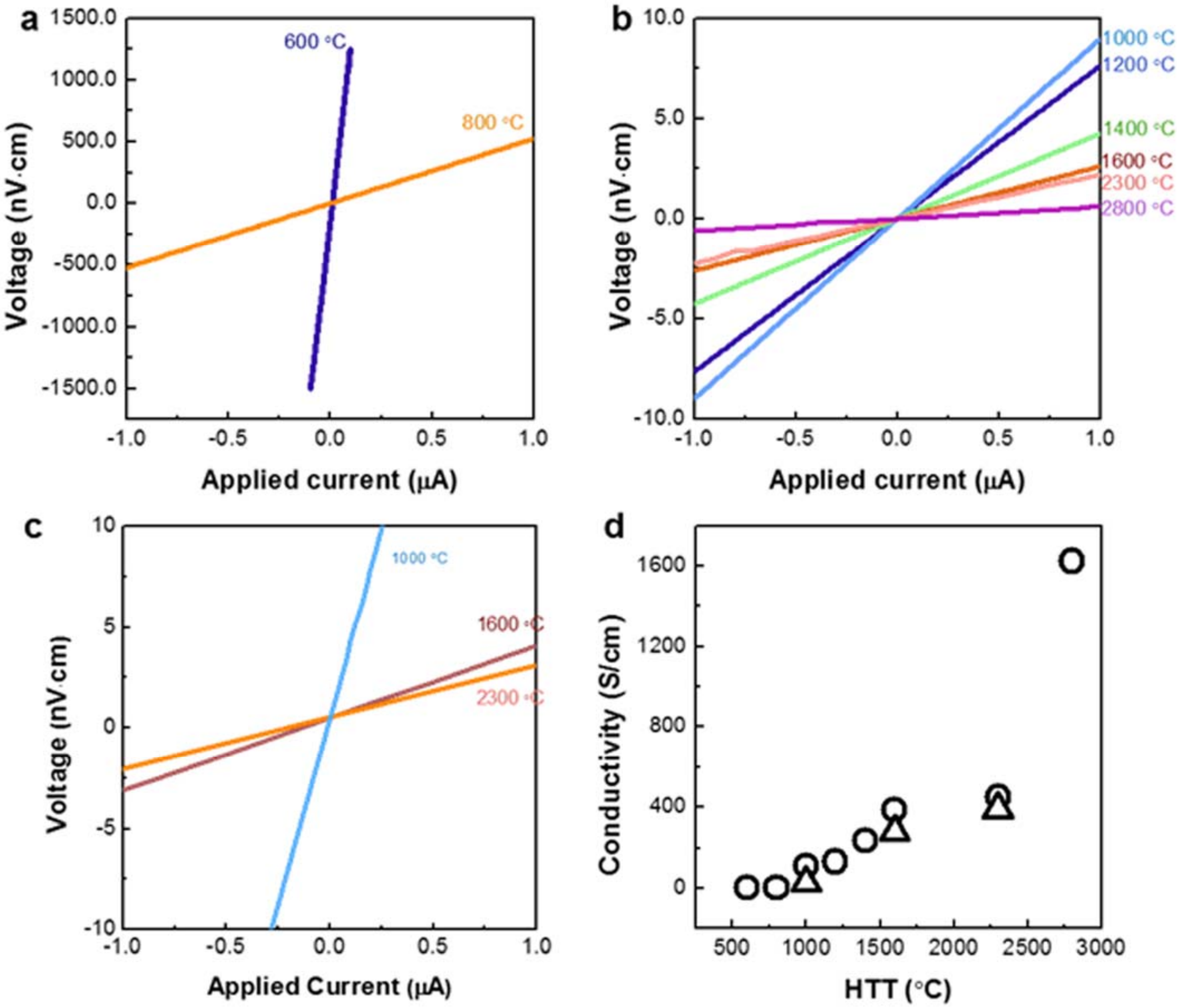

Supplementary Figure 11. The electrical transport properties of silk protein prepared at high temperature. The voltage as a function of the applied current of worm silk (**a**,**b**) and spider silk (**c**) heated to temperatures in the range 600–2,800°C. This was obtained using a conventional four-probe method. (**d**) The HTT dependence of the differential conductivity of the worm silk (circle) and spider silk (triangle).

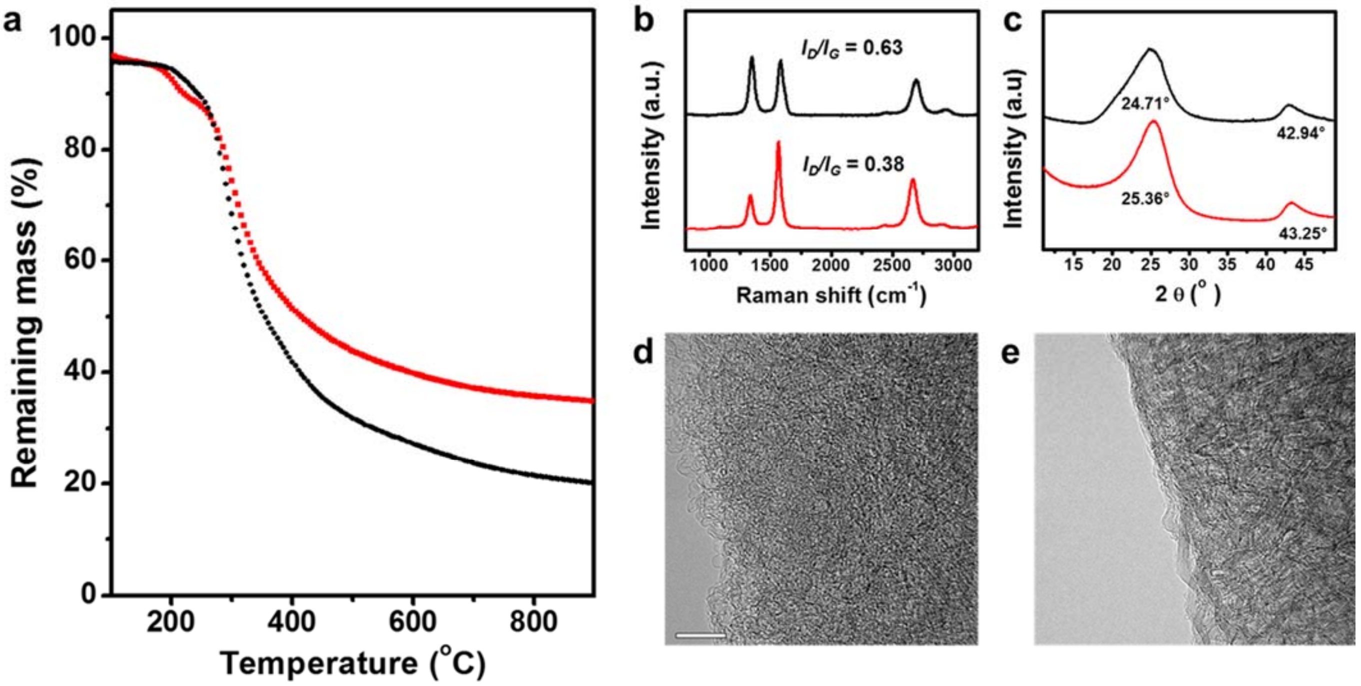

Supplementary Figure 12. Carbonisation of regenerated silk proteins with and without crystallisation. (**a**) TGA data, (**b**) Raman spectra, and (**c**) XRD patterns of the as-prepared RSF (black curve) and crystallised RSF (red curve) heated to 2,800ºC. FE-TEM images of (**d**) the as-prepared RSF and (**e**) the crystallised RSF heated to 2,800ºC. The scale bar in **d** represents 100 µm and refers to **e**.

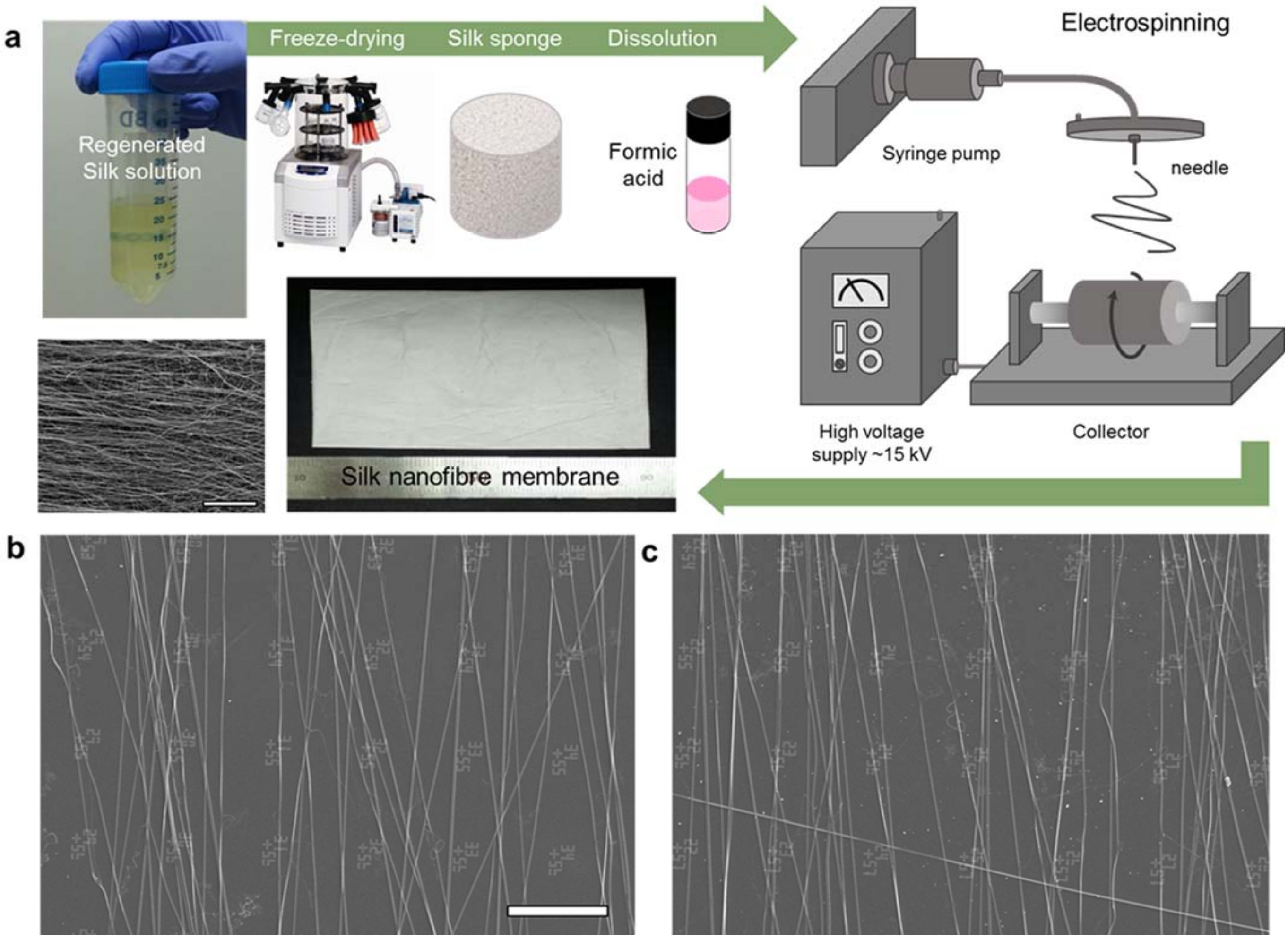

Supplementary Figure 13. One-dimensional carbon nanofibres from regenerated silk protein. (**a**) Schematic diagram showing the electrospinning process for silk nano-fibres. (**b**) FESEM images of the as-spun silk nano-fibres. (**c**) FESEM images of carbonised silk nano-fibres, which were heated to 800ºC. The scale bar in **a** represents 200 µm. The scale bar in **b** represents 100 µm and refers to **c**.

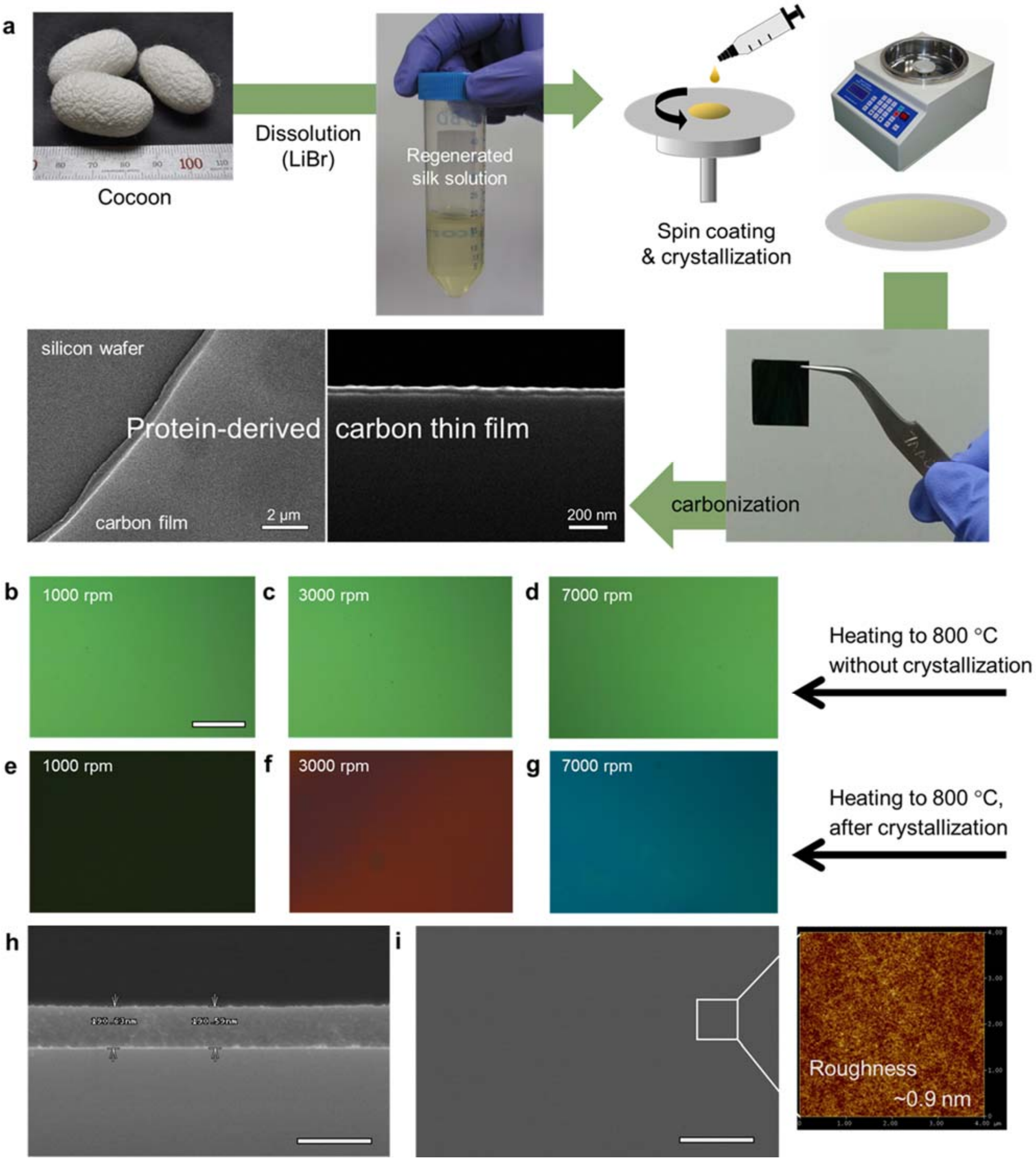

Supplementary Figure 14. Two-dimensional carbon thin films from regenerated silk protein. (**a**) Schematic diagram of the preparation of the silk-protein-derived carbon thin film *via* a wet process. (**b–g**) Optical microscope images of the as-prepared and β-sheet induced regenerated silk protein films with different spin-coating speeds following carbonisation at 800ºC. The scale bar in **b** represents 10 μm and refers to **c-g.** (**h**) FESEM images of fractured carbon thin films spin-coated at 1000, 3000, and 7000 rpm. Scale bar, 500 nm. (**i**) FESEM and AFM images of carbon thin film spin-coated at 7000 rpm. Scale bar, 25 μm.

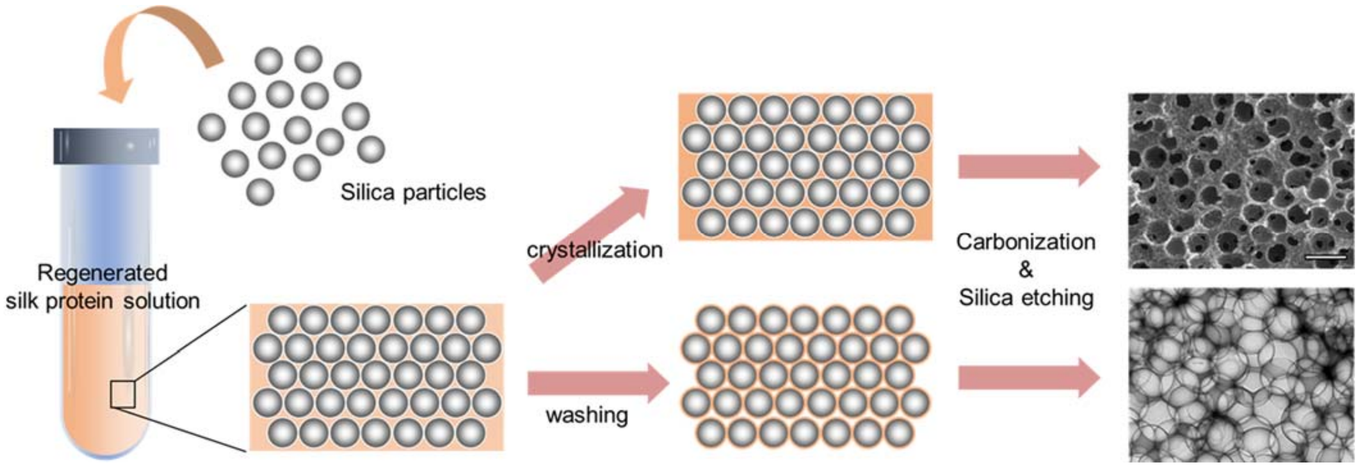

Supplementary Figure 15. Schematic diagram showing the fabrication process for the three-dimensional nanospheres and porous carbon material. Scale bar, 500 nm.

Supplementary Table

Supplementary Table 1

The carbon, oxygen, and nitrogen contents of the silk-protein-derived carbon heat-treated at various temperatures, as determined from XPS measurements.

	atomic composition (%)											
**HTT**	**raw**	**250ºC**	**300ºC**	**350ºC**	**400ºC**	**600ºC**	**800ºC**	**1000ºC**	**1200ºC**	**1600ºC**	**2300ºC**	**2800ºC**
**C**	**63.7**	**61.0**	**61.6**	**78.2**	**80.1**	**81.2**	**83.1**	**90.8**	**92.1**	**94.3**	**96.7**	**97.4**
**N**	**15.6**	**17.9**	**18.1**	**10.6**	**8.4**	**8.6**	**7.4**	**3.7**	**2.4**	**2.1**	**0.4**	**0.4**
**O**	**20.7**	**21.2**	**20.3**	**11.2**	**11.5**	**10.2**	**9.5**	**5.5**	**5.5**	**3.7**	**2.9**	**2.2**

Supplementary notes

Supplementary note 1. Supplementary Fig. 4a shows TGA analysis and Supplementary Fig. 4b shows DSC analysis of the silk proteins with heating rates of 2, 5, 10, and 20ºC min^{-1} in an inert atmosphere. Following an initial mass loss of approximately 5 wt.% during TGA analysis at temperatures below 150 ºC (due to the evaporation of absorbed water)[1], substantial thermal degradation of the protein samples began at around 250 ºC. All of the protein samples exhibited similar forms of degradation curve (i.e. rapid mass loss from 250 to 350ºC and continuous mass loss above 350ºC), and the residual mass was negatively correlated with the heating rate (the residual masses at 800ºC were 34.8, 33.3, 28.1 and 26.0% with heating rates of 2, 5, 10, and 20 ºC min^{-1}, respectively). These results suggest that a more rapid heating rate promotes more breaking of protein chains, with subsequent gasification. The DSC thermograms for the silk protein samples reveal stronger endothermic effects, corresponding to the breaking of covalent bonds and the formation of flue gases, as the heating rate increased. However, we may assume that the thermally degraded polypeptide chains are transformed not only into flue gases, but also into thermally stable ring structures (via exothermic reactions). The DSC and TGA results support this. The relatively slow heating rates exhibit smaller endothermic peaks in the DSC data, and an endotherm curve was not observed with a heating rate of 2ºC min^{-1}, as well as larger residual masses in the TGA curves. Supplementary Fig. 4c shows a simple energy diagram, which can explain the rapid mass loss as simultaneous reactions of the thermal degradation of the silk protein, followed by a transition to volatile oligomers or thermally stable ring structures.

Supplementary note 2. Supplementary Fig. 5a shows the sample holder used to measure the current–voltage (*I-V*) characteristics at high temperatures. The Pt wires were used as the signal lines, and were connected with the Au wires that were in contact with the sample. Samples were mounted between alumina plates as shown in the right panel of Supplementary Fig. 5a. Supplementary Fig. 5b shows the *I-V* characteristics of worm silk fibres prepared at different HTTs in the range 250–400°C, which were obtained using a conventional two-probe method. We observed an increase in the current as the HTT increased. To compare the variation in conductivity as a function of the HTTs, the conductivity σ_{1V} was obtained from the current at an applied voltage of 1.0 V (see Supplementary Fig. 5c). When the silk fibres were heated to

250°C, we find σ_{IV} = 1.4 × 10^{-4} S cm^{-1}, compared with σ_{IV} = 2.7 × 10^{-6} S cm^{-1} for the untreated worm silk fibre. (For the pristine silk fibre, the current was approximately 20 pA at 1.0 V). The conductivity increased monotonically up to an HTT of 320°C (where σ_{IV} = 3.3 × 10^{-4} S cm^{-1}). At 350°C, the conductivity increased abruptly to 1.2 × 10^{-3} S cm^{-1}, which is threefold that at 320°C. A significant increase in conductivity at 350°C was also found from *in situ* measurements using a custom-built holder in an N_2 atmosphere. Supplementary Fig. 6a shows σ_{IV} obtained from the *I-V* characteristics of the worm silk, and Supplementary Fig. 6b shows the *I-V* characteristics of spider silk fibres. In both cases, we observed that the colour changed as the temperature increased beyond 250°C, and that σ_{IV} abruptly increased at 350°C. This is consistent with the *ex situ* data shown in Supplementary Fig. 5

Supplementary note 3. The protein chain linkage via peptide bonding is flexible, and can form a diverse range of molecular conformations. However, the supramolecular interactions, such as hydrogen bonding, π-interactions between aromatic groups, and van der Waals forces, result in various local conformations of the polypeptide (i.e. a secondary structure) such as α-helices, β-sheets and turns or loops. The β-sheet conformation is characterised by numerous assemblies of intra- and/or inter-chain hydrogen bonds between adjacent peptide blocks. The highly conserved primary sequence in the silk protein determines the dominant β-sheet secondary structure. The GX repeats of the amino acid sequence in the crystalline domain lead to inter-unit hydrogen bonds, and develop to form a two-dimensional folded chain, which is aligned anti-parallel to the β-sheet conformation. In addition, amino acids with small side chains, such as glycine and alanine, facilitate inter-sheet stacking to form three-dimensional nano-crystals due to van der Waals interactions[2]. A typical β-sheet crystallite silk fibroin is a rectangular lattice with a coordinate system defined with the *x*-axis oriented along the amino acid side chains, the *y*-axis in the direction of the hydrogen bonds, and the *z*-axis along the direction of the peptide bonds; the lattice constants are a = 0.938 nm, b = 0.949 nm, and c = 0.698 nm[3]. The microstructure of the native silk fibroin fibres is a block copolymer structure of stiff antiparallel β-sheet blocks aligned with the fibre axis, and dispersed in soft amorphous segments and empty space. Therefore, the carbon layers from β-sheet crystals are observed with parallel to the fibre axis.

Supplementary note 4. In order to evaluate the development of the microstructure of silk protein derived carbon materials, first order region of Raman spectra (1100-1800 cm^{-1}) was deconvolved, and the integral intensity of the representative characteristic peaks for the carbon materials centered at ~1350 (*D* band) and ~1580 (*G* band) cm^{-1} was calculated (Supplementary Fig. 8). From the silk protein sample heat-treated at 400ºC, the *D* and *G* bands were clearly revealed, i.e., the sp^2 carbon structure with a few nanometer carbon layer was formed by heat treatment of the silk protein. The integral intensity ratio of the *D* to *G* bands (I_D/I_G) was maintained until 800ºC at approximately 2.6 with L_a value of ~2 nm. The intensity ratio of the Raman spectra decreased, and the *D* and *G* bands become narrower between 800~1000ºC, indicating the development of a sp^2 carbon structure, which can be interpreted as a result of the elimination of residual oxygen and nitrogen spices as confirmed by XPS results (Supplementary Table 1). With HTT above 1000ºC, the crystalline sp^2 carbon structure developed systematically, and a highly developed graphitic structure with an average of L_a = 16 nm was formed as the HTT increased to 2,800ºC

Supplementary methods

Preparation of regenerated silk fibroin (RSF) solution

To extract the glue-like sericin proteins and impurities, silkworm cocoons were boiled for 25 min in an aqueous solution of 0.02-M Na_2CO_3 (i.e. a degumming process). Following washing with DI water several times, the resulting silk fibroin fibres were dried at room temperature for 3 days. Regenerated silk protein solutions were prepared as described elsewhere[4]. Briefly, degummed silk fibroin fibres were dissolved in an aqueous solution of 9.3-M LiBr (99%; Sigma-Aldrich, St. Louis, MO, USA) at 60°C for 6 hours. The silk protein solution was dialysed in water using a Slide-a-Lyzer dialysis cassette (MWCO 3500; Pierce) for 2 days. The final concentration of the silk fibroin in the aqueous solution was approximately 8 wt%.

Electrospun nano-fibres

Electro-spinning was employed to fabricate silk protein nano-fibres. Following freezing for 6 hours at -80 ºC, the frozen regenerated silk solution was subjected to lyophilisation at -50 ºC and 0.045 mbar for 72 hours.

The resulting cylindrical silk sponge was dissolved in 98% formic acid (Junsei Chemical Co. Japan) with mild stirring for 1 day under ambient conditions. The mass fraction of silk protein (relative to formic acid) was 10%. The silk protein solution was filled in a plastic syringe and the syringe was connected to a metal needle that was 0.514 mm diameter. A rotating disk with an angular velocity of 1500 rpm surrounded by aluminium foil was placed 15 cm from the needle and used to collect the electrospun silk nano-fibres. A voltage x of 15 kV was applied between the metal needle and the aluminium foil. The electro-spinning process was performed at room temperature with a relative humidity of less than 40%.

Crystallisation of regenerated silk protein

The regenerated silk proteins were formed into a β-sheet configuration using thermal drawing. Following drying for 3 days in an oven at 30°C, the electrospun silk protein membrane was cut into 50 x 10 mm strips, and immersed in DI water for a seconds. The softened silk protein specimen was elongated to 100 mm (without breaking) in the axial direction at a rate of 10 mm min^{-1} and thermal annealed for 1 hour at 200°C using a universal testing machine (UTM, Model No. 4200, Instron, USA) equipped with a thermal chamber.

Carbonisation of silk nano-fibres

The as-prepared and elongated silk nano-fibres were heated to 800ºC with controlled heating schedules in a tubular furnace under an argon atmosphere (minimum purity, 99.9990%; gas flow, 100 cm^3 min^{-1}). The heating schedule is an important factor in determining the quality of carbon materials obtained from a carbon precursor. The carbonisation process was as follows. The silk nano-fibres were rapidly heated to 150ºC, and this temperature was maintained for 2 hours to remove residual water. Rapid heating (40 ºC min^{-1}) was then used to increase the temperature from 150 to 350ºC to reduce the effects of thermal recrystallisation of the RSF nano-fibres (which occurs at temperatures in the range 190–220ºC). The samples were maintained at 350 ºC for 3 hours to induce the formation of the conjugated carbon structure, and the samples were heated to the desired final temperatures at a rate of 5°C min^{-1}, followed by a 2-hour isotherm at that temperature.

Fabrication of carbon thin films

A simple spin-casting method was used to fabricate thin films of regenerated silk aqueous solutions on a silicon wafer. To fabricate polymeric films with a uniform thickness, the silk solution was diluted to 2.0 mg mL^{-1}, and approximately 200 μL of the silk solution were used to fabricate each film. Spin rates of 1000, 3000 and 7000 rpm were used to form films with various thicknesses. The resulting thin films were soaked in methanol for 3 hours, and then heated to 800ºC in a tubular furnace under an argon atmosphere (minimum purity, 99.9990%; gas flow, 100 cm^3 min^{-1}). The carbonisation process was identical to that used for the nano-fibres.

Fabrication of hollow carbon nano-spheres

A 20-g aqueous dispersion containing 200 mg of silica nanospheres was prepared separately via ultrasonication. The dispersion was then poured into the regenerated silk solution and stirred vigorously for 30 min. The mixture was frozen using liquid nitrogen, and freeze-dried using a lyophiliser at -50°C and 0.045 mbar for 72 hours. The silk-coated silica nanosphere cryogels were dispersed in water and sonicated for 5 min; the dispersion then was rapidly poured into a methanol bath. After 6 hours, the mixture was vacuum-filtered and dried in a vacuum oven at 30°C for 24 hours. The isolated particulates were carbonised as described for the nano-fibres. Following carbonisation, the resulting particulates were treated with 30-wt.% hydrofluoric acid solution to remove the silica particles, and washed with DI water and ethanol.

Carbon microstructure

The microstructure of the carbon materials was characterised using Raman spectra and XRD data. The Raman spectra for the carbon materials exhibited two representative bands; i.e. an E_{2g} vibration mode attributed to the stretching vibration in the graphite layers, known as the *G* band, which occurred at around 1580 cm^{-1}, and the A_{1g} breathing mode of the sixfold aromatic ring near the basal edge corresponding to the disorder, termed the *D* band, which occurred at around 1350 cm^{-1}. The ratio of integral intensity of the *D*

band to that of the G band, I_D/I_G, is commonly used to characterise the crystallite dimensions of the carbon layers L_a using the Tuinstra and Koenig equation[5]; i.e.

$$\frac{I_D}{I_G} = \frac{C(\lambda)}{L_a}$$

where $C(\lambda)$ is a constant that depends on the laser wavelength ($C(\lambda)$ = 4.4 nm for a the 514-nm laser used here). From the XRD results, the interlayer distance (i.e. d-spacing, d_{002}) can be calculated using the Bragg equation; i.e.

$$n\lambda = 2d_{002} \sin\theta$$

where n is the integral number, λ is the wavelength and θ is the maximum angle of the diffraction peak.

Electrical conductivity

The electrical conductivity of the carbonised silk protein fibres was investigated as a function of the HTT using a current-voltage (I-V) characteristics measured at 300 K using a conventional two-probe method for the samples heat-treated at temperatures up to 400°C, and using a and four-probe method for the samples annealed at temperatures in the range 600–2800°C. Silver paint (DuPont 4929N) contacts were attached to gold wires (0.01 inches in diameter). With the two-probe measurements, the I-V characteristics were measured using a semiconductor characterisation system (4200-SCS; Keithley). With the four-probe method, the I-V characteristics were measured using a current source (6220 DC current source; Keithley) and a nanovoltmeter (2182 nanovoltmeter; Keithley).

Supplementary References

1. Ling, S. et al. Synchrotron FTIR microspectroscopy of single natural silk fibers. *Biomacromolecules* **12**, 3344-3349 (2011).

2. Hardy, J. G., Römer, L. M. & Scheibel, T. R. Polymeric materials based on silk proteins. *Polymer* **49**, 4309-4327 (2008).

3. Karakutuk, I., Ak, F. & Okay, O. Diepoxide-triggered conformational transition of silk fibroin: formation of hydrogels. *Biomacromolecules* **13**, 1122-1128 (2012).

4. Jin, H. J., & Kaplan, D. L. Mechanism of silk processing in insects and spiders. *Nature* **424**, 1057-1061 (2003).

5. Tuinstra, F. & Koenig, J. L. Raman spectrum of graphite. *J. Chem. Phys.* **53**, 1126-1130 (1970).
